# Supplementary material for: Learning curve and surgical outcome of robotic assisted colorectal surgery with ERAS program
Source: Sci Rep. 2022 Nov 29;12:20566. doi: 10.1038/s41598-022-24665-w (PMC9709162; doi:10.1038/s41598-022-24665-w)
Supplement: Supplementary file 1 — Supplementary Information 1. [file 41598_2022_24665_MOESM1_ESM.zip › LSC Raw data-Ñ[▒K/111/N_ñw╝╨░O▒Kñσ.pdf]

W52 / W52

臺 中 榮 民 總 醫 院
手術室護理記錄

[redacted]

基本資料

- 室別： 二醫手術室-36室，手術科別： CRS，手術類別： 常規，日期： 2020/12/24
- 進入時間： 1430 ； 麻醉開始時間： 1430 ； Time Out時間： 1453 ； 劃刀時間： 1454 ； 手術結束時間： 1840 送出時間： 1850 ；
- 麻醉方式： GE
  診斷：IC VALVE polyps FAILURE TO EMR(TRY MONPOLAR ONLY SURGERY)
  手術名稱：Laparoscopic right colectomy and anastomosis
  手術局部用藥：nil
  術前預防性抗生素：
  - 抗生素名稱一： Veterin 1GM IVD/AR
  - 時間： 1458
  - 上次簽章: 吳明柔

  ，追加預防性抗生素：
- 手術醫師： 蔣鋒帆
- 麻醉醫師： VS 蔡佩馨
-
  - 刷手護士A ： 許育庭，時間： 1430，刷手護士B ： 鄭宜芳，時間： 1430，接替刷手一： 王毓均，時間： 1500，接替刷手二： 胡貞伶，時間： 1700
  - 流動護士A ： 吳明柔，時間： 1430，接替流動一： 王惟瑩，時間： 1730，接替流動二： 吳明柔，時間： 1800
- 更多接替及流動護士： 接替刷手三： 張仟品，時間： 1800

皮膚狀況

A-V Shunt： 無
術前： 完整
  記錄者： 上次簽章: 吳明柔
術後： 完整
  記錄者：

手術準備

手術臥式 單‧ ‧手術臥式

-
  - 手術臥式： 仰臥
  - 使用附件： 溫毯 38 ℃，矽膠枕頭，其它： 頭架
  - 皮膚準備： 10%Aq Betadine，Hibiscrub，2% Chlorhexidine
  - 電燒： 使用電燒: 電極片位置： 右大腿，記錄者： 上次簽章: 吳明柔

止血帶T： 無

植入物

無

標本送檢

- 細菌培養 無
- 細胞學 無
- 冰凍切片 無
- 病理標本
  組織名稱一：A COLON
- 截肢 無

標本交付 標本： 壹 袋，標本： 零 盒，標本： 零 瓶，細胞學： 零 管，Culture Tube： 零 管，被交付醫師： CR 陳映菁，

- 記錄者： 吳明柔
- 時間： 1900

傷口術後敷料

傷口敷料： 傷口敷料： 無

- 請於圖上標示部位：

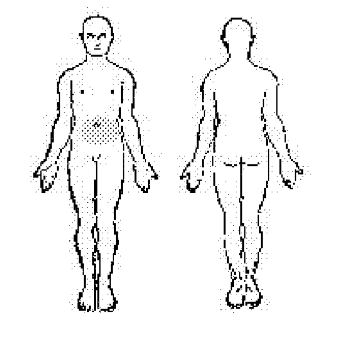

- 傷口敷料： 紗布

導管

導管	天數	導管資訊	插入日期	插入時間	更換日期	拔除日期	拔除時間	拔除原因	預計拔除/更換	預計拔除/更換日期
Penrose Drain(1)	1	號碼:12 mm.; 部位:吻合處;	2020/12/24	1750						
Foley(1)	1	號碼:16 Fr.; 部位:膀胱;	2020/12/24	1440						

用物計數

- 手術中出血量： MINIMAL c.c.
- 手術中尿量： 550 c.c.
-
  - 其它： 無

用物計數： 多切口同時縫合

- 1.器械： 正確，2.縫針： 正確，3.刀片： 正確，4.紗布： 正確，5.腹部墊： 未使用，6.紗球： 未使用，7.PEANUTS ： 未使用，8.紗條： 未使用，9.絲帶： 正確，10.針頭： 未使用，11.Vessel loop： 未使用，12.Boots： 未使用，13.Jelco： 未使用，14.棉條： 未使用，
  - 第一次：
    - 刷手護士 ： 胡貞伶
    - 流動護士： 上次簽章: 王惟瑩
    - 時間： 1745

  ,
  - 第二次：
    - 刷手護士 ： 胡貞伶
    - 流動護士： 上次簽章: 王惟瑩
    - 時間： 1755

  ,
  - 第三次：
    - 刷手護士 ： 胡貞伶
    - 流動護士： 上次簽章: 王惟瑩
    - 時間： 1800

備註

備註：NIL

- 記錄者： 吳明柔 上次簽章: 吳明柔
- 時間： 1900

填寫護理人員 吳明柔

正式摘要

W52 / W52

doctorRank:B
